# Sumoylation of TCF21 downregulates the transcriptional activity of estrogen receptor-alpha

**DOI:** 10.18632/oncotarget.8354

**Published:** 2016-03-25

**Authors:** Xiang Ao, Shujing Li, Zhaowei Xu, Yangyang Yang, Min Chen, Xiao Jiang, Huijian Wu

**Affiliations:** ^1^ School of Life Science and Biotechnology, Dalian University of Technology, Dalian 116024, Liaoning, People's Republic of China; ^2^ School of Life Science and Medicine, Dalian University of Technology, Panjin 114221, Liaoning, People's Republic of China

**Keywords:** sumoylation, TCF21, ERα, transcriptional activity, proliferation

## Abstract

Aberrant estrogen receptor-α (ERα) signaling is recognized as a major contributor to the development of breast cancer. However, the molecular mechanism underlying the regulation of ERα in breast cancer is still inconclusive. In this study, we showed that the transcription factor 21 (TCF21) interacted with ERα, and repressed its transcriptional activity in a HDACs-dependent manner. We also showed that TCF21 could be sumoylated by the small ubiquitin-like modifier SUMO1, and this modification could be reversed by SENP1. Sumoylation of TCF21 occurred at lysine residue 24 (K24). Substitution of K24 with arginine resulted in complete abolishment of sumoylation. Sumoylation stabilized TCF21, but did not affect its subcellular localization. Sumoylation of TCF21 also enhanced its interaction with HDAC1/2 without affecting its interaction with ERα. Moreover, sumoylation of TCF21 promoted its repression of ERα transcriptional activity, and increased the recruitment of HDAC1/2 to the *pS2* promoter. Consistent with these observations, sumoylation of TCF21 could inhibit the growth of ERα-positive breast cancer cells and decreased the proportion of S-phase cells in the cell cycle. These findings suggested that TCF21 might act as a negative regulator of ERα, and its sumoylation inhibited the transcriptional activity of ERα through promoting the recruitment of HDAC1/2.

## INTRODUCTION

Breast cancer is the most frequently diagnosed malignant tumor and the leading cause of cancer related death in women worldwide [1]. The incidence rate of breast cancer is gradually increasing due to changes in reproductive factors and increased screening intensity [2]. The occurrence of breast cancer is contributed by multiple factors, such as family history, reproductive factors, lifestyle, alcohol consumption and exposure to estrogen [3–6]. Among them, exposure to estrogen is recognized as the main contributor to breast carcinogenesis. Estrogen exerts its effects through binding to the estrogen receptors (ERs) ERα and ERβ, which belong to the nuclear receptor superfamily. After binding with estrogen, the receptors dimerize and bind to DNA at the estrogen response elements (EREs) of downstream target genes and associate with coactivators or corepressors to regulate the expression of these genes. Numerous coactivators have been shown to interact with ERα, such as cAMP-response element binding protein (CREB)-binding protein, p300, steroid receptor coactivator-1 (SRC-1), SRC-2 and SRC-3 [7]. Fewer corepressors have been reported to date, which include NCoR, SMRT and histone deacetylases (HDACs) [8]. ERα is associated with tumor initiation and development in 70–80% of breast-cancer patients [9]. Clinically, ERα is considered as a good prognostic factor in breast cancer and a major target for endocrine therapy [10].

Transcription factor 21 (TCF21), also known as capsulin, epicardin or Pod1, belongs to the basic-helix-loop-helix (bHLH) family of transcription factors [11, 12]. It was first cloned from mouse embryo. TCF21 is widely expressed in mesenchymal cells at the epithelial-mesenchymal interaction sites during the development of urogenital, cardiovascular, respiratory, and gastrointestinal systems [13]. It plays crucial roles in cell fate and differentiation during the development of organs, including the heart, vasculature, lung, kidney, and spleen [14, 15]. Loss of TCF21 leads to spleen, kidney, and lung abnormalities and neonatal lethality [13, 14]. TCF21 is recognized as a candidate tumor suppressor and has been reported to be epigenetically inactivated due to aberrant methylation of the *TCF21* gene promoter in a wide range of malignancies, including metastatic melanoma [16], head and neck [17, 18], lung [18–23], gastric [24] and urological cancers [25]. In human adrenocortical tumor cells, TCF21 inhibits the expression of endogenous SF-1 and the SF-1 target gene *StAR* through binding to the E-box sequence of *SF-1* promoter [13]. In renal cancer, TCF21 is a target protein of miR-21 and its down-regulation increases the invasive ability of Caki-1 cells [26]. However, little is known about the role of TCF21 in human breast cancer. Several members of the bHLH family have been reported to interact with ERα and regulate its function through acting as coregulators, such as amplified in breast cancer 1 (AIB1) [27] and circadian locomotor output cycles kaput (CLOCK) [7]. Both ERα and androgen receptor (AR) have the classical nuclear receptor structure [28]. TCF21 can interact with AR and inhibit its transactivation through promoting the recruitment of HDAC1 [28]. We therefore wanted to know whether TCF21 can directly interact with ERα and inhibit its activity.

Post-translational modifications (PTMs), such as phosphorylation, methylation, acetylation, ubiquitination and sumoylation, are important mechanisms for regulating protein functions [29]. At present, few studies have focused on the PTM of TCF21 other than its phosphorylation [14]. SUMO (small ubiquitin-like modifier) is a small protein that is covalently attached to a lysine residue of its target proteins via C-terminal di-glycine in the sequence *Ψ*KXE/D (*Ψ* represents a large hydrophobic amino acid and X represents any amino acid). Sumoylation involves several steps and three well-known enzymes called activating enzyme (E1), conjugating enzyme (E2), and ligases (E3). Similar to phosphorylation, sumoylation is a reversible process, and SUMO can be removed from the SUMO-protein conjugate by SUMO-specific proteases (SENPs) [30, 31]. Sumoylation plays important roles in protein regulation, such as altering protein subcellular localization, protein stability, protein–protein interaction and transcriptional activity. Many nuclear proteins with important roles in cellular processes have been shown to be subject to sumoylation, such as differentiated embryo-chondrocyte expressed gene 1 (DEC1) [32], G-protein pathway suppressor 2 (GPS2) [33] and aryl hydrocarbon receptor (AhR) [34]. Analysis of the amino acid sequence of TCF21 revealed two potential SUMO acceptor sites, K24 and K65, and we therefore speculated that TCF21 may be a target of sumoylation.

In this report, we showed that TCF21 negatively regulated the transcriptional activity of ERα in a HDAC1/2-dependent manner. We also showed that TCF21 could be sumoylated, and the sumoylation of TCF21 was essential to its negative regulation of ERα. This negative regulation of ERα led to a reduction in breast cancer cell proliferation. Our data have given new insight into the involvement of TCF21 in estrogen-signaling pathway, with ERα as its key interacting transcription factor.

## RESULTS

### TCF21 interacts with ERα in breast cancer cells

**Figure 1:**
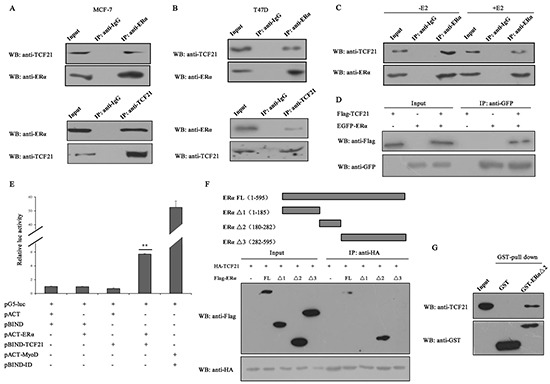
Interaction between TCF21 and ERα **A–B.** MCF-7 and T47D cells were subjected to IP with anti-ERα antibody followed by Western blot with anti-TCF21 and anti-ERα antibodies or vice versa. IP carried out with anti-IgG antibody was used as control. **C.** MCF-7 cells treated with or without E2 were subjected to IP using anti-ERα antibody followed by Western blot with anti-TCF21 antibody. IP carried out with anti-IgG antibody was used as control. **D.** HEK 293T cells transfected with Flag-TCF21 only, EGFP-ERα only, or with Flag-TCF21 plus EGFP-ERα were subjected to IP using anti-GFP antibody followed by Western blot with anti-Flag antibody. **E.** Interaction between TCF21 and ERα as demonstrated by mammalian two hybrid system. TCF21 and ERα were expressed from pBIND-TCF21 and pACT-ERα, respectively, whereas the empty vectors pACT and pBIND were used as controls, as indicated by the pG5-luc reporter in MCF-7 cells. Cells transfected with pBIND-ID and pACT-MyoD were used as positive control. Luciferase activity was measured 36 h after transfection. The luc activity level of cells transfected with pG5-luc, pACT and pBIND was set to 1. Data are the means ± S.Ds of three experiments. ‘**’ indicates significantly different from cells transfected with pACT and pBIND at the *P* <0.01 level. **F.** HEK 293T cells were transfected with HA-TCF21 and Flag-tagged full-length ERα (FL), ERα Δ1, ERα Δ2, or ERα Δ3. The cells were subjected to IP using anti-IgG or anti-HA antibody followed by Western blot with anti-Flag antibody. **G.** Interaction between TCF21 and ERα Δ2 in vitro. Purified His-TCF21 was incubated with immobilized GST- ERα Δ2 or GST alone. The bound proteins were subjected to Western blot assay. All experiments were repeated at least three times. Data are the mean ± SDs of three independent experiments.

TCF21 is a member of bHLH family of transcription factors, and it has been reported to interact with AR and inhibit its function [28]. As ERα and AR possess similar and classical nuclear receptor structure, we speculated that TCF21 may interact with ERα. In order to investigate this possibility, immunoprecipitation (IP) experiment was conducted in two different ERα-positive breast cancer cell lines, MCF-7 and T47D, using either anti-TCF21 or anti-ERα antibody. A positive interaction between endogenous TCF21 and ERα was observed in MCF-7 and T47D cells (Figure 1A and 1B). The effect of estrogen on the interaction between endogenous TCF21 and ERα was examined in MCF-7 cells following treatment with or without 17β-estradiol (E2). In presence of E2 treatment, the interaction between endogenous TCF21 and ERα was weakened compared to that in absence of E2 treatment (Figure 1C). The positive interaction between exogenous TCF21 and ERα was also obtained when the same IP experiment was performed in HEK 293T cells transfected with Flag-TCF21 and EGFP-ERα (Figure 1D). Moreover, mammalian two hybrid assay further confirmed the interaction between TCF21 and ERα. Transactivation by pBIND–TCF21 was evident when co-expressed with pACT–ERα, which expressed an ERa-fusion protein (Figure 1E).

To established which region of ERα might be involved in mediating its interaction with TCF21, HEK 293T cells were transfected with HA-TCF21 together with Flag-tagged full-length ERα (ERα FL) or either one of the three truncated forms of ERα (ERα Δ1 contained ligand-independent transcriptional activation function 1 domains, ERα Δ2 contained DNA-binding domain, and ERα Δ3 contained ligand-binding domain). The transfected cells were subjected to IP carried out with anti-HA antibody, followed by Western blot with anti-Flag antibody. Positive interaction was obtained only between TCF21 and ERα FL or Δ2, but not between TCF21 and ERα Δ1 or Δ3 (Figure 1F), indicating that the region containing DNA-binding domain mediated the interaction between ERα and TCF21. GST pull-down assay using purified GST-ERα Δ2 and His-TCF21 in vitro further confirmed the interaction between ERα and TCF21 (Figure 1G).

### TCF21 represses the transcriptional activity of ERα

**Figure 2:**
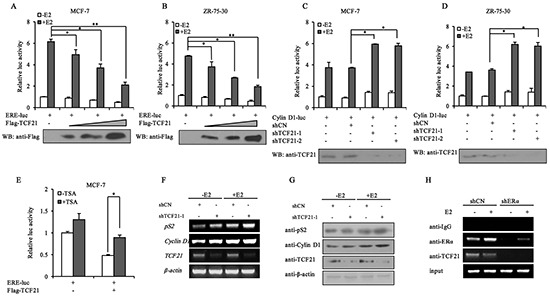
Effect of TCF21 on the transcriptional activity of ERα **A–B.** MCF-7 and ZR-75-30 cells were transfected with ERE-luciferase and Flag-TCF21. Luciferase activity was detected either with or without pre-treatment of the cells with 10 nM E2 for 16 h. For comparison, the ERE-luc activity level of control cells was set to 1. ‘*’ and ‘**’ indicate significantly different from cells transfected with ERE-luc only at the *P*<0.05 and *P*<0.01 level, respectively. **C–D.** MCF-7 and ZR-75-30 cells were transfected with Cyclin D1-luciferase and shCN, shTCF21-1 or shTCF21-2. Luciferase activity was detected with or without pre-treatment of the cells with 10 nM E2 for 16 h. For comparison, the Cyclin D1-luc activity level of control cells was set to 1. ‘*’ indicates significantly different from cells transfected with Cyclin D1-luc and shCN at the *P*<0.05 level. **E.** MCF-7 cells were transfected with ERE-luc and Flag-TCF21. Luciferase activity was detected either with or without 10 nM TSA for 20 h. For comparison, the ERE-luc activity level of control cells was set to 1. ‘*’ indicates significantly different from cells treated with TSA at the P<0.05 level. **F.** MCF-7 cells were transfected with shTCF21-1. Cells pre-treated with or without 10 nM E2 for 16 h were subjected to PCR to measure the mRNA levels of *pS2* and *Cyclin D1*. **G.** MCF-7 cells transfected with shTCF21-1 were treated with or without 10 nM E2 for 16 h. The samples were subjected to Western blot analysis with the indicated antibodies. **H.** ChIP experiment showing the binding of TCF21 and ERα to ERE of the *pS2* promoter in MCF-7 cells. MCF-7 cells transfected with shERα were treated with or without 10 nM E2 for 16 h. The cells were subjected to IP with anti-IgG, anti-ERα, or anti-TCF21 antibody. All experiments were repeated at least three times. Data are the means ± SDs of three independent experiments.

TCF21 is a transcription repressor, and it exerts its function through interacting with a corepressor complex [14]. Therefore, the interaction of TCF21 with ERα was expected to have a negative effect on the transcriptional activity of ERα. To investigate this possibility, luciferase reporter assay was performed using a reporter gene construct consisting of the estrogen responsive element-luciferase (ERE-luc). Following E2 treatment, MCF-7 and ZR-75-30 cells co-transfected with ERE-luc and TCF21 showed significant reduction in luciferase activity, compared to those transfected with ERE-luc only (Figure 2A and 2B). Furthermore, the reduction in luciferase activity was dependent on the dose of TCF21, and occurred with or without E2 treatment. We next tested the inhibitory effect of TCF21 on ERα by using a known ERα-target gene, *Cyclin D1*, which also contains EREs in its promoter. Luciferase activity significantly increased in MCF-7 cells when the endogenous *TCF21* gene was silenced by shTCF21, compared to that in MCF-7 cells without silencing of TCF21 (Figure 2C). Similar results were obtained in ZR-75-30 cells (Figure 2D).

TCF21-mediated repression of AR transactivation is known to involve the activity of HDAC1. In addition, HDAC2 has been shown to associate with TCF21 in proepicardial cells [14]. Therefore, trichostatin A (TSA), an inhibitor of HDACs, was used to examine whether HDACs were also involved in TCF21-mediated repression of ERα. MCF-7 cells co-transfected with ERE-luc and TCF21 showed significant reduction in luciferase activity compared to those transfected with ERE-luc only (Figure 2E), and the inhibition of ERα transcriptional activity by TCF21 was significantly attenuated by TSA, indicating that HDACs were involved in TCF21-mediated inhibition of ERα transcriptional activity. Moreover, we examined the effect of TCF21 on the expression of the well-established ERα target genes (*pS2* and *Cyclin D1*) in MCF-7 cells. PCR and Western blot assays showed that knockdown of endogenous TCF21 increased the mRNA and protein levels of both *pS2* and *Cyclin D1* (Figure 2F and 2G).

To further examine the corepressor function of TCF21 on ERα, ChIP assay was conducted in MCF-7 cells transfected with shERα in presence or absence of E2. Chromatins prepared from MCF-7 cells were immunoprecipitated with anti-IgG, anti-ERα, or anti-TCF21. In the case of MCF-7 cells that were transfected with shCN, most of the endogenous TCF21 was bound to the *pS2* promoter, whereas knockdown of ERα in these cells resulted in the loss of binding between TCF21 and the *pS2* promoter (Figure 2H). Taken together, these results suggested that TCF21 probably acted as a corepressor and inhibited the transcriptional activity of ERα.

### TCF21 is modified by SUMO1 and desumoylated by SENP1

**Figure 3:**
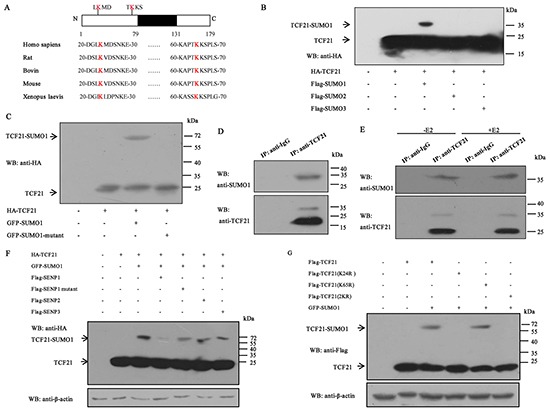
Sumoylation of TCF21 **A.** Schematic representation of the primary structures of TCF21 and the predicted sumoylation sites along with the structures of TCF21 in different species. **B.** HEK 293T cells transfected with HA-TCF21 and Flag-SUMO1, 2 or 3 were subjected to Western blot with anti-HA antibody. **C.** HEK 293T cells co-transfected with GFP-SUMO1 or GFP-SUMO1 mutant and HA-TCF21 were subjected to Western blot with anti-HA antibody. **D.** HEK 293T cells were subjected to IP using anti-TCF21 antibody or anti-IgG antibody followed by Western blot using anti-SUMO1 or anti-TCF21 antibody. **E.** MCF-7 cells treated with or without E2 were subjected to IP with anti-TCF21 antibody followed by Western blot with anti-SUMO1 antibody. **F.** HEK 293T cells were transfected with different combinations of constructs as indicated, followed by Western blot with anti-HA antibody. No NEM was added in the cell extracts. **G.** HEK 293T cells were transfected with Flag-tagged wild-type or mutant TCF21(K24R, K65R or 2KR) and GFP-SUMO1, and individual cell extracts were subjected to Western blot with anti-Flag antibody. All experiments were repeated at least three times.

Several members of the bHLH family such as AIB1, CLOCK and DEC1 have been shown to be subject to sumoylation [7, 30, 32]. However, whether TCF21 can be modified by SUMO has not been determined, although it is subject to phosphorylation [14]. The protein sequence of TCF21 was analyzed, and two potential sumoylation sites (K24 and K65) were found, which were conserved in different species (Figure 3A). To test whether TCF21 can also be sumoylated, HEK 293T cells were transfected with both HA-tagged TCF21 and one of the three Flag-tagged SUMO expressing plasmids. TCF21 was shown to be efficiently modified by SUMO1 as demonstrated by the soluble fraction of cell lysate (Figure 3B). Further confirmation of TCF21 sumoylation was achieved by Western blot analysis of the extract of HEK 293T cells that were transfected with HA-TCF21 and GFP-SUMO1 or GFP-SUMO1/GA, which is a SUMO mutant that can't bind to the substrate due to a C-terminal diglycine substitution (GG to GA). Western blot analysis revealed that only cells transfected with TCF21 and wild-type SUMO1 showed a positive migrating band (Figure 3C). In addition, immunoprecipitated TCF21 from HEK 293T cells was detected by anti-SUMO1 antibody. The size of this band corresponded to that of SUMO-conjugated TCF21 (Figure 3D). We next examined the effect of estrogen on the sumoylation of endogenous TCF21 in MCF-7 cells following treatment with or without E2. No significant difference was observed in the presence or absence of E2 treatment (Figure 3E).

Three SENPs (SENP1, 2 and 3) are present in the cell nucleus. To determine which SENP could reverse the sumoylation of TCF21, HEK 293T cells were co-transfected with HA-TCF21 and GFP-SUMO1 with Flag-SENP1, Flag-SENP1 mutant, SENP2, or SENP3. Only cells transfected with SENP1 showed reduced level of migrating band compared to cells transfected with SENP1 mutant, SENP2 and SENP3 (Figure 3F), indicating that SENP1 mediated the desumoylation of TCF21. We next tested whether K24 and K65 are required for the sumoylation of TCF21. HEK 293T cells were co-transfected with GFP-SUMO1 and Flag-tagged wild type or mutant TCF21(K24R, K65R, or 2KR). Western blot analysis showed that changing K65 to arginine (K65R) did not affect the sumoylation of TCF21, whereas changing either K24(K24R) or both K24 and K65 to arginine (2KR) appeared to abolish sumoylation of TCF21 (Figure 3G), indicating that K24 was the unique site of sumoylation for TCF21. These data showed that TCF21 could be modified by SUMO1, SENP1 mediated the desumoylation of TCF21, and K24 was the unique sumoylation site of TCF21.

### Sumoylation stabilizes TCF21 protein and increases its interaction with HDAC1/2

**Figure 4:**
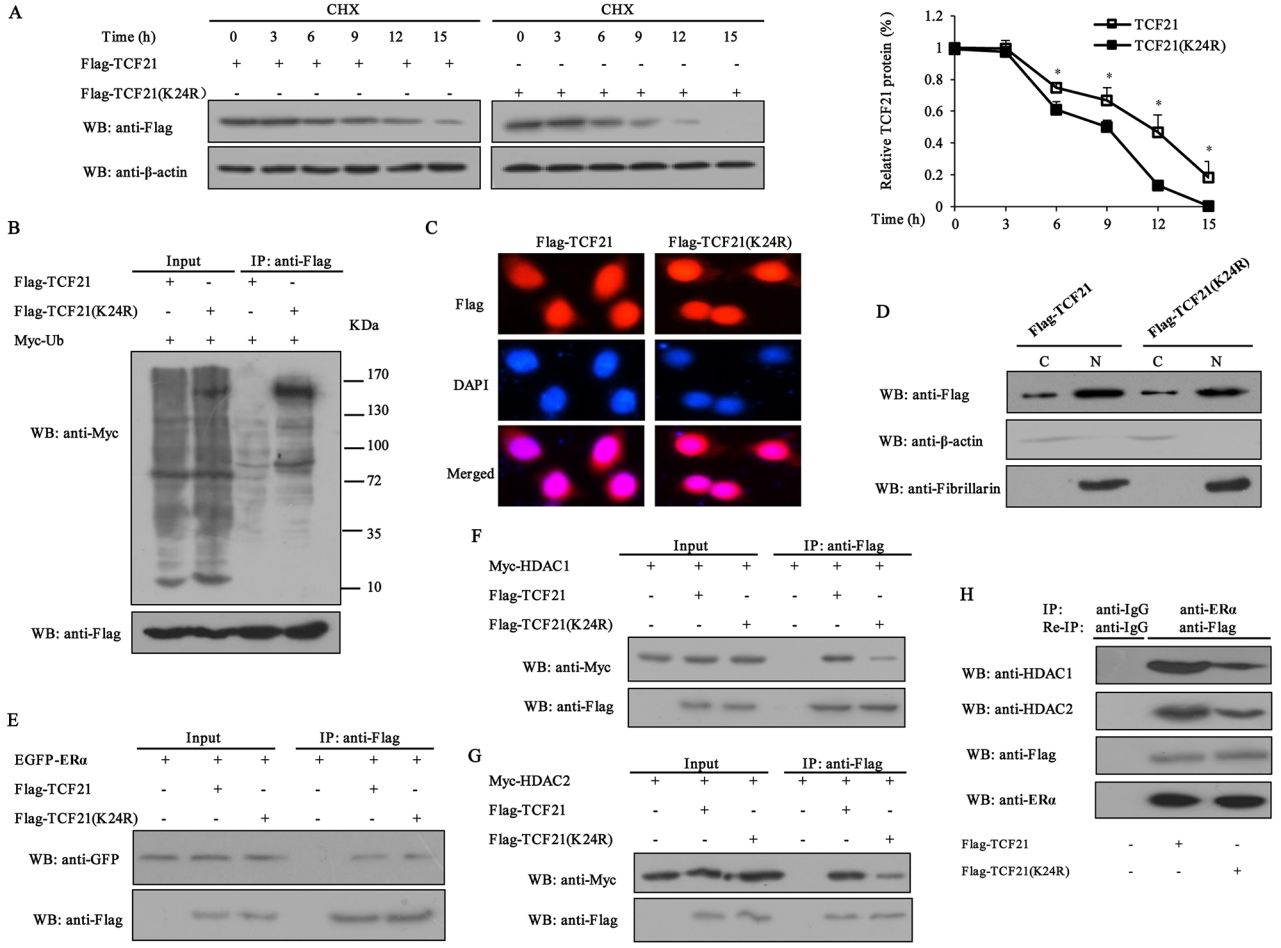
Effect of sumoylation on the half-life of TCF21, subcellular localization and its interaction with ERα and HDAC1/2 **A.** MCF-7 cells transfected with Flag-tagged wild-type or mutant TCF21(K24R) were treated with 10 mg/ml CHX at the indicated time periods. Cell lysate was subjected to Western blot assay. The graph shows the relative intensity of the TCF21 band at different time points. The level of TCF21 protein in control cells was set to 1. Data shown in the graphs are the means ± SDs of three independent experiments. ‘*’ indicates significantly different from cells transfected with mutant TCF21 (K24R) at the *P*<0.05 level. **B.** HEK 293T cells transfected with Myc-Ub and Flag-tagged wild-type TCF21 or mutant TCF21(K24R) were collected, and then subjected to IP using anti-Flag antibody followed by Western blot with anti-Myc or anti-Flag antibody. **C.** MCF-7 cells transfected with Flag-tagged wild-type or mutant TCF21(K24R) were stained with rabbit anti-Flag antibody (red) and then counterstained with DAPI (blue) for nucleus detection. **D.** MCF-7 cells were transfected with Flag-tagged wild-type or mutant TCF21(K24R). Cytosolic and nuclear fractions were subjected to Western blot using anti-Flag antibody. Fibrillarin and β-actin were measured to monitor the efficiency of nuclear and cytosolic preparations, respectively. **E.** HEK 293T cells transfected with EGFP-ERα and Flag-tagged wild-type or mutant TCF21(K24R) were collected, and then subjected to IP using anti-Flag antibody followed by Western blot with anti-GFP or anti-Flag antibody. **F.** HEK 293T cells transfected with Myc-HDAC1 and Flag-tagged wild-type or mutant TCF21(K24R) were collected, and then subjected to IP using anti-Flag antibody followed by Western blot with anti-Myc or anti-Flag antibody. **G.** HEK 293T cells transfected with Myc-HDAC2 and Flag-tagged wild-type or mutant TCF21 (K24R) were collected, and then subjected to IP with anti-Flag antibody followed by Western blot with the anti-Myc or anti-Flag antibody. **H.** MCF-7 cells transfected with Flag-tagged wild-type or mutant TCF21(K24R) were subjected to IP using anti-ERα antibody and re-IP with anti-Flag antibody followed by Western blot with anti-HDAC1, anti-HDAC2, anti-Flag or anti-ERα antibody.

Given that sumoylation is usually involved in the regulation of protein stability, we determined the effect of sumoylation on TCF21 stability by treating MCF-7 cells with the protein translation inhibitor cycloheximide (CHX) for different time periods following their transfection with Flag-TCF21 or Flag-TCF21(K24R). The result showed that the half-life of wild-type TCF21 was about 11 h, whereas that of TCF21 (K24R) was only about 9 h (Figure 4A), indicating that sumoylation could increase the half-life of TCF21 through stabilizing the protein. The effect of K24R mutation on the ubiquitination of TCF21 was examined by IP assay. The result showed that the ubiquitination of TCF21 (K24R) significantly increased compared to wild-type TCF21 (Figure 4B), indicating that the attachment of SUMO1 to TCF21 probably masked the regions recognized by the ubiquitin machinery. TCF21 is mainly localized in the nucleus, but can also be found in the cytoplasm. Sumoylation is known to affect the subcellular localization of a protein [35, 36]. Next, we determined the effect of sumoylation on the nuclear translocation of TCF21. MCF-7 cells were transfected with Flag-tagged wild-type TCF21 or its mutant K24R and then subjected to immunofluorescence staining. No significant differences in nuclear and cytoplasmic distributions of TCF21 were observed between wild-type and mutant (Figure 4C). Similar results were obtained when the cytosolic and nuclear fractions of the cells were subjected to Western blot analysis (Figure 4D).

Sumoylation was reported to influence the interaction of proteins and the recruitment of HDACs [37, 38]. Therefore, IP assay was conducted to examine the effect that TCF21 sumoylation might have on its interaction with ERα and HDACs. The results showed that sumoylation of TCF21 did not affect its interaction with ERα (Figure 4E), but enhanced its interaction with HDAC1/2 (Figure 4F and 4G). Since TCF21 could interact with both ERα and HDAC1/2, we speculated that an ERα-TCF21-HDAC1/2 complex might exist. Re-IP and Western blot were performed to investigate this possibility. A positive band was detected when extract of MCF-7 cells that were transfected with Flag-TCF21 or Flag-TCF21 (K24R) was probed with anti-HDAC1 or anti-HDAC2 antibody, and this band was weakened in cells transfected with mutant TCF21 (K24R), compared to the cells transfected with wild-type TCF21 (Figure 4H). These results indicated the existence of an ERα-TCF21-HDAC1/2 complex and the sumoylation of TCF21 were required for the interaction between TCF21 and HDAC1/2.

### Sumoylation of TCF21 represses the transcriptional activity of ERα

**Figure 5:**
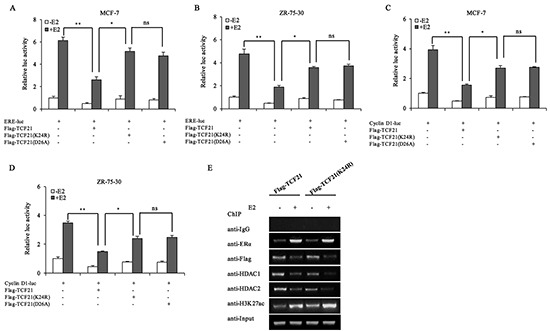
Effect of TCF21 sumoylation on the transcriptional activity of ERα **A–B.** MCF-7 and ZR-75-30 cells were transfected with the indicated plasmids. Luciferase activity was detected either with or without pre-treatment of the cells with 10 nM E2 for 16 h. For comparison, the ERE-luc activity level of control cells was set to 1. ‘**’ indicates significantly different from cells transfected with ERE-luc and Flag-TCF21 at the *P*<0.01 level. ‘*’ indicates significantly different from cells transfected with ERE-luc and mutant TCF21 (K24R) at the *P*<0.05 level. ‘ns’ indicates not significant. **C–D.** MCF-7 and ZR-75-30 cells were transfected with Cyclin D1-luc, Flag-TCF21, Flag-TCF21 (K24R), or Flag-TCF21(D26A). Luciferase activity was detected either with or without pre-treatment of the cells with 10 nM E2 for 16 h. For comparison, the level of Cyclin D1-luc activity in control cells was set to 1. ‘**’ indicates significantly different from cells transfected with Cyclin D1-luc and Flag-TCF21 at the *P*<0.01 level. ‘*’ indicates significantly different from cells transfected with Cyclin D1-luc and mutant TCF21(K24R) at the *P*<0.05 level. ‘ns’ indicates not significant. **E.** MCF-7 cells were transfected with Flag-tagged wild-type TCF21 or mutant TCF21(K24R). Cells pre-treated with or without 10 nM E2 for 16 h were subjected to ChIP to examine the recruitment of ERα, TCF21, HDAC1/2 and acetyl-H3K27 to the *pS2* promoter. All experiments were repeated at least three times. Each bar represents the mean ± SDs of three independent experiments.

TCF21 interacted with HDAC1/2, and exerted its corepressor function in a HDAC-dependent manner, and sumoylation appeared to play a role in enhancing the interaction between TCF21 and HDAC1/2. Therefore, sumoylation of TCF21 was expected to have a negative effect on the transcriptional activity of ERα. The effect of TCF21 sumoylation on the transactivation activity of ERα was examined by luciferase reporter assay. Lysine residue can be modified by several PTMs, such as acetylation, ubiquitination and sumoylation. To clarify that alteration of the transactivation activity of ERα was induced by TCF21 sumoylation instead of other modifications, another TCF21 sumoylation mutant TCF21 (D26A) besides TCF21 (K24R) was used. As expected, in presence of E2 treatment, MCF-7 and ZR-75-30 cells transfected with ERE-luc and wild-type TCF21 showed significant reduction in luciferase activity compared to those transfected with ERE-luc only. However, when the cells were transfected with mutant TCF21(K24R) or TCF21(D26A), the inhibition exerted by TCF21 against the transactivation activity of ERα was attenuated, and TCF21(D26A) showed similar effect with TCF21(K24R) (Figure 5A and 5B), indicating that the sumoylation of TCF21 inhibited the transcriptional activity of ERα. We obtained similar results using Cyclin D1-luc reporter gene (Figure 5C and 5D). To elucidate the molecular mechanism by which TCF21 sumoylation may inhibit ERα transactivation, ChIP assay was conducted in MCF-7 cells transfected with wild-type TCF21 or mutant TCF21 (K24R), following treatment with or without E2. When MCF-7 cells were transfected with the mutant TCF21, binding between ERα and *pS2* promoter was not affected, whereas binding between HDAC1/2 and *pS2* promoter was decreased, leading to higher accumulation of acetylated-H3K27 in *pS2* promoter (Figure 5E). Taken together, these results demonstrated that sumoylation of TCF21 might repress the transcriptional activity of ERα through recruiting HDAC1/2.

### Effect of TCF21 sumoylation on the growth of breast cancer cells

**Figure 6:**
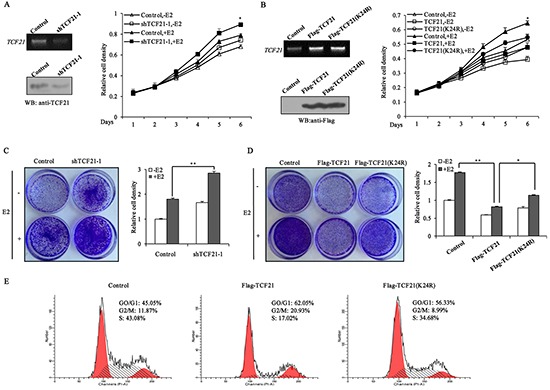
Sumoylation of TCF21 inhibits the growth of breast cancer cell lines **A.** MCF-7 cells were stably transfected with control vector, or shTCF21-1. MTT assay was conducted either with or without pre-treatment of the cells with 10 nM E2 for the indicated times. The mRNA and protein levels of TCF21 were examined by PCR and Western blot assay. **B.** MCF-7 cells were stably transfected with control vector, Flag-TCF21or Flag-TCF21(K24R). MTT assay was conducted either with or without pre-treatment of the cells with 10 nM E2 for the indicated times. The mRNA and protein levels of TCF21 were examined by PCR and Western blot assay. **C.** MCF-7 cells stably transfected with control vector, or shTCF21-1 were stained with crystal violet after 8 days of growth (left panel), and the corresponding quantitative analyses are shown on the right panel. For comparison, the number of control cells was set to 1. ‘**’ indicates significantly different from cells transfected with shTCF21-1 at the *P*<0.01 level. **D.** MCF-7 cells stably transfected with control vector, Flag-TCF21or Flag-TCF21(K24R) were stained with crystal violet after 8 days of growth (left panel), and the corresponding quantitative analyses are shown on the right panel. For comparison, the number of control cells was set to 1. ‘**’ indicates significantly different from cells transfected with Flag-TCF21 at the *P*<0.01 level. ‘*’ indicates significantly different from cells transfected with Flag-TCF21 (K24R) at the *P*<0.05 level. **E.** MCF-7 cells were transfected with control vector, or with Flag-tagged wild-type or mutant TCF21(K24R). The cell-cycle distribution of MCF-7 cells was examined by flow cytometry analysis after 16 h of growth in presence of 10 nM E2. All experiments were repeated at least three times. Data are the means ± SDs of three independent experiments.

ERα is known to promote the proliferation of breast cancer cells. Since sumoylation of TCF21 could repress the transcriptional activity of ERα, and down-regulate its target genes expression, we speculated that sumoylation of TCF21 may also inhibit the proliferation of ERα-positive breast cancer cells. MTT assay showed that the growth of TCF21 knockdown cells was more than control cells, both in absence and presence of E2 treatment (Figure 6A). However, cells stably transfected with wild-type TCF21 showed less growth than cells stably transfected with mutant TCF21(K24R) (Figure 6B). These results suggested that the growth of these cells was regulated by TCF21-modulated transcription factors (including ERα), and this process might depend on the sumoylation status of TCF21. In addition, crystal violet staining assay was conducted to further confirm the inhibition of TCF21 on the proliferation of ERα-positive breast cancer cells. TCF21 knockdown cells produced more colonies than control cells in absence and presence of E2 (Figure 6C). However, cells stably transfected with wild-type TCF21 produced less colonies than cells stably transfected with mutant TCF21(K24R) (Figure 6D). The effect of TCF21 sumoylation on the cell-cycle was also investigated. MCF-7 cells transfected with wild-type TCF21 resulted in an overall decrease in the percentage of S-phase cells, with a corresponding increase in the percentage of cells in the G0/G1 phase, compared to control cells (Figure 6E). Overexpression of mutant TCF21 had little effect on the cell cycle. Taken together, these results suggested that TCF21 could repress the growth of breast cancer cells, and this process was dependent on TCF21 sumoylation.

## DISCUSSION

Breast cancer is one of the most prevalent causes of cancer death among women. Despite the continuous biomedical research efforts, breast cancer is still a major public health problem. About 50–80% of breast tumors are ERα-positive [9]. ERα has been recognized as a favorable prognostic factor and a major target of endocrine therapy for breast cancer treatment [10]. Aberrant ERα signaling has been shown to be correlated with the initiation and development of breast cancer [39]. Therefore, further insight into the detailed mechanism involved in the regulation of ERα function is important for us to understand the pathogenesis of ERα-positive breast cancer, and to facilitate the development of more effective breast cancer treatment strategies. In this study, we showed that in human ERα-positive breast cancer cell lines, TCF21 participated in the ERα-signaling pathway through acting as a negative regulator of ERα, and the interaction between TCF21 and ERα could inhibit the transcriptional activity of ERα. TCF21 was a direct substrate of sumoylation, and its sumoylation could repress the transcriptional activity of ERα through promoting the recruitment of HDAC1/2.

The bHLH transcription factors are characterized by the helix-loop-helix (HLH) domain, and they play crucial roles in the differentiation and proliferation of cells [14, 40]. TCF21 belongs to class II bHLH transcription factors [28], and is considered as a tumor suppressor, but the detailed mechanism is still unclear. TCF21 was reported to inhibit the proliferation and invasion of lung cancer cells [18]. It also can inhibit the invasion of metastatic melanoma and renal cancer cells through transactivating *KISS1* expression [16, 26]. In prostate cancer, TCF21 interacts with AR and inhibits its transactivation through the recruitment of HDAC1 [28]. Our data from IP, mammalian two hybrid system and GST pull-down assay clearly demonstrated that TCF21 could also interact with ERα, and the region containing the DNA-binding domain of ERα mediated the interaction between the two proteins (Figure 1). Moreover, subsequent reporter gene and ChIP assays showed that TCF21 repressed the transactivation of ERα, and was recruited to *pS2* promoter in an ERα-dependent manner. These results suggested that TCF21 may act as a corepressor of ERα. Our data also demonstrated that HDACs were major contributors in TCF21-mediated repression of ERα transcriptional activity (Figure 2E). This is consistent with its role in mediating the inhibitory effect of TCF21 on AR [28].

Post translational modification of proteins endows proteins with multiple functions [41]. Sumoylation can alter the subcellular localization of a protein or its stability, protein-protein interaction and transcriptional activity of transcription factors [42]. We demonstrated that TCF21 could be sumoylated by SUMO1 but not by SUMO2/3 (Figure 3B–3E), and identified K24 as the unique site of TCF21 sumoylation (Figure 3G). Protein modification by sumoylation is a dynamic process, which is governed by both sumoylation and desumoylation [43]. Our data showed that desumoylation of TCF21 was mediated by SENP1 rather than SENP2 and 3 (Figure 3F). When K24 was mutated, the half-life of TCF21 was reduced from about 11 h to about 9 h (Figure 4A). The attachment of SUMO to a protein often alters the surface of the protein and might cause a conformational change at critical interfaces, thereby affecting its ability to interact with other proteins [30, 33]. The sumoylation site of TCF21 was located in the N-terminal (K24) region. Analysis of the primary structure of TCF21 in different species of organisms revealed K24 as a conserved residue. We have shown that TCF21 interacted with both ERα and HDAC1/2, suggesting that sumoylation might regulate its interaction with ERα or HDAC1/2. We found that sumoylated TCF21 did not affect its interaction with ERα (Figure. 4E), but contributed to its association with HDAC1/2 (Figure 4F and 4G) and therefore increased its ability to inhibit the transcriptional activity of ERα, a property that would be favorable to its regulation of cell functions. These results corresponded to the data from ChIP assay, which showed that TCF21 sumoylation promoted the recruitment of HDAC1/2 to the *pS2* promoter, but had no effect on the recruitment of ERα (Figure 5E). Aberrant ERα signaling contributes to the initiation and development of breast cancer by up-regulating its target genes, such as *Cyclin D1* and *pS2* [44–46]. HDACs can inhibit the transcriptional activity of ERα [47]. We demonstrated that sumoylation of TCF21 could enhance its interaction with HDAC1/2, which eventually inhibited the transcriptional activity of ERα (Figure 5A–5D).

ERα plays an important role in promoting the proliferation of breast cancer cells. Overexpression of ERα in breast cancer cells lines results in the induction of cell growth, whereas knockdown of ERα expression by siRNA blocks estradiol-stimulated cell proliferation[39]. Given that TCF21 could act as a negative regulator of ERα, it was expected to play a role in ERα-mediated cell proliferation. TCF21-knockdown cells showed more growth than control cells. Moreover, overexpression of either wild-type or mutant TCF21(K24R) could inhibit the growth of breast cancer cells, but mutant TCF21 showed weaker ability to repress the growth of breast cancer cells than wild-type TCF21 (Figure 6A and 6B). In fact, TCF21 could inhibit the growth of MCF-7 in the presence or absence of E2. A similar trend was observed in the crystal-violet-staining assay (Figure 6C and 6D). Moreover, wild-type TCF21 appeared to inhibit the cell cycle progression of MCF-7 cells, whereas this ability was attenuated when the K24 site of TCF21 was mutated (Figure 6E). These results provided evidence that sumoylation was necessary for TCF21 to fulfill its repressive role in the regulation of ERα.

In conclusion, we showed here for the first time that TCF21 negatively regulated the function of ERα through promoting the recruitment of HDAC1/2. TCF21 was a direct substrate of sumoylation and that sumoylation of TCF21 was necessary for the negative regulation of ERα by TCF21. This negative regulation of ERα would disrupt the growth of ERα-positive breast cancer cells, which would mean a reduction in cell proliferation and the spread of cancer cells. Although we have shown here that TCF21 may function as a corepressor in the ERα-signaling pathway and sumoylation of TCF21 was crucial in this progress, the detailed mechanism of TCF21 in regulating cell functions is still inconclusive. Therefore, elucidating the relationship between TCF21 and ERα network will further uncover the molecular mechanism of TCF21 in regulating cell functions, and this may provide better insight for conceiving a way to combat breast cancer.

## MATERIALS AND METHODS

### Plasmids and antibodies

Flag-ERα, EGFP-ERα, ERE-luc, Cyclin D1-luc, Myc-HDAC1 and Myc-HDAC2 have been described in our previous studies [7, 48, 49]. Human TCF21 was amplified from a human cDNA library using the following primers: 5′-GCATGAATTCTATGTCCACCGGCTCCCTCA-3′ (forward) and 5′-CGATCTCGAGTCAGGA-CGCGGTGGTTCCA-3′ (reverse), and the amplified TCF21 DNA fragment was inserted into the expression vector pcDNA3.1-3×Flag and pcDNA3.1-HA at the *Eco*RI and *Xho*I sites. Flag-TCF21 mutants (K24R, D26A, K65R and 2KR) were generated using a site-directed mutagenesis kit according to the manufacturer's instruction (Stratagene, La Jolla, CA, USA). Flag-tagged truncated ERα (Δ1, Δ2, and Δ3) was designed as previously described [50], and constructed according to standard PCR-based cloning procedures using Flag-ERα as a template. PCR fragments were inserted into pcDNA3.1-3×Flag at *Eco*RI and *Xho*I sites (Δ1 and Δ3) or at *Bam*HI and *Xho*I sites (Δ2).

Rabbit anti-Flag and mouse anti-Flag antibodies were obtained from Sigma. Mouse anti-ERα and anti-GST antibodies were purchased from Millipore. Mouse anti-HA, mouse anti-GFP, rabbit anti-GFP rabbit, and rabbit anti-H3K27ac antibodies were obtained from GeneTex. Rabbit anti-ERα, anti-IgG, anti-HDAC1, and anti-HDAC2 antibodies, and mouse anti-Fibrillarin and anti-β-actin antibodies were obtained from Santa Cruz Biotechnology. Rabbit anti-TCF21 and anti-SUMO1 antibodies, and anti-rabbit IgG VeriBlot for IP secondary antibody and anti-mouse IgG VeriBlot for IP secondary antibody were obtained from Abcam. Rabbit anti-Cyclin D1 and anti-pS2 antibodies were obtained from BBI Life Sciences. CHX and G418 were obtained from Sigma. Hygromycin B was obtained from Roche Molecular Biochemical. TSA was obtained from Beyotime. N-Ethylmaleimide (NEM) was obtained from Pierce.

### Cell culture and transfection

Human embryo kidney cell lines HEK 293T, human breast cancer cell lines T47D and cells have been used in our previous studies [51, 52]. MCF-7 and ZR-75-30 cells were obtained from the cell bank of the Shanghai branch of Chinese Academy of Sciences. HEK 293T and MCF-7 were maintained in Dulbecco's modified Eagle's medium (DMEM, Invitrogen) supplemented with 10% fetal bovine serum (Hyclone), whereas T47D cells were maintained in Roswell Park Memorial Institute (RPMI) 1640 medium supplemented with 10% fetal bovine serum (Hyclone) and 0.2 U/ml insulin. ZR-75-30 cells were maintained in Roswell Park Memorial Institute (RPMI) 1640 medium supplemented with 10% fetal bovine serum. All cells were incubated at 37°C in presence of 5% CO_2_. The cells were transiently or stably transfected with appropriate plasmids using Lipofectamine 2000 (Invitrogen) according to the company's specification. Corresponding empty vectors were used to guarantee the same total amount of plasmids for all parallel groups. For 17β-estradiol (E2) stimulation experiments, cells were cultured in phenol red-free medium containing 2% charcoal-stripped fetal bovine serum (Gibco) for 24 h, followed by treatment with or without 10 nM E2 for 16 h. For stable transfection, MCF-7 cells were selected by 1000 mg/ml G418 or 300 mg/ml hygromycin B after transient transfection. The medium was replaced with fresh medium containing G418 or hygromycin B every two days.

### Immunoprecipitation and western blot assay

Cells were lysed in a cold hypotonic buffer (50 mM Tris-HCl [pH=7.4], 150 mM NaCl, 0.1% SDS, 1% NP-40, 0.5% sodium deoxycholate and 100-fold diluted protease inhibitor mixture). The cell lysate was centrifuged at 10000×*g*/4°C for 10 min. After that, the supernatant was extracted and incubated with the appropriate antibody at 4°C for overnight, followed by addition of protein A-Sepharose or protein G-Sepharose and further incubation at 4°C for another 8 h. After centrifugation at 5000×*g*/4°C for 10 min, the supernatant was removed, and then the precipitate was washed twice with Washing Buffer I (50 mM Tris–HCl [pH=7.5], 150 mM NaCl, 1% NP-40 and 0.05% NaDC) and once with Washing Buffer II (50 mM Tris–HCl [pH=7.5], 500 mM NaCl, 0.1% NP-40, and 0.05% NaDC). After washing, the precipitate was resuspended in SDS-PAGE sample-loading buffer, and boiled at 100°C for 5 min, and then subjected to SDS-PAGE. After electrophoresis, protein bands in the gel were transferred onto polyvinylidene fluoride (PVDF) membrane (Millipore), and then probed with the appropriate primary and secondary antibodies. Immunoblot data were quantified by scanning the bands of interest and plotted them as relative density of gray scale. For sumoylation assay, 30 mM of N-Ethylmaleimide (NEM, Pierce), a SUMO protease inhibitor, was added to the lysis buffer to block the SENP activity by acting as a general alkylating agent that modifies the active site cysteine. Re-IP was conducted as previously described [31].

### GST pull-down assay

GST-ERα (Δ2) was prepared by cutting Flag-ERα (Δ2) with *Bam*HI and *Xho*I, and the fragment was then inserted into pGEX-4T3 (Amersham Pharmacia). The GST and GST-fusion protein were expressed in BL21(DE3) (Takara), and purified by Pierce GST Spin Purification Kit (Thermo scientific). His-TCF21 was prepared by cutting Flag-TCF21 with *Eco*RI and *Xho*I, and then the fragment was inserted into pET32a(+) (Novagen). His-TCF21 protein was expressed in BL21 and purified by Ni-Agarose His-tagged Protein Purification Kit (CW Biotech). GST pull-down assay was performed using a Pierce GST Protein Interaction Pull-Down Kit (Thermo scientific).

### Mammalian two hybrid assay

The CheckMate TM mammalian two-hybrid system was obtained from Promega (Madison, USA). ERα was subcloned into *Bam*HI*–Eco*RV cut pACT, and TCF21 was subcloned into *Bam*HI*-Kpn*I cut pBIND.

### Immunofluorescence staining

Cells were cultured on coverslips for 24 h. After that, the cells were fixed in 4% paraformaldehyde for 15 min at room temperature, and then permeabilized with cold anhydrous methanol at −20°C for 40 min. The fixed cells were blocked with 0.8% bovine serum albumin (BSA) at 4°C for 1 h, and then incubated with anti-flag antibody at 4°C for overnight. After washing with PBS, the coverslips were incubated with anti-rabbit secondary antibody for 1 h at room temperature. The coverslips were then washed with PBS and incubated with 4′,6-diamidino-2-phenylindole (DAPI).

### RNA interference

shTCF21 was constructed by DNA vector-based shRNA synthesis using pRNATU6.1/Hygro (GenScript, Piscataway, NJ) vector. The sequences used for TCF21 silencing were selected according to siGENOME Human TCF21 (6943) siRNA (GE Dharmacon): 5′-CCAGCTACATCGCCCACTT-3′ (shTCF21-1) and 5′-AACCTGACGTGGCCCTTTA-3′ (shTCF21-2). The sequences of the negative control shRNA and shERα have been described in our previous study [53].

### Luciferase reporter assay

Transcription activity was examined by a luciferase assay system. Cells were seeded into 24-well plates at 1×10^5^ per well, and cultured for 24 h. The cells were then transfected with the appropriate plasmids using Lipofectamine 2000 according to the company's specification. Twenty four hours after transfection, the medium was replaced with phenol red-free medium containing 2% charcoal-stripped fetal bovine serum (Gibco) for 24 h, followed by treatment with or without 10 nM E2 for 16 h. The cells were then subjected to luciferase and Renilla activity assays according to the manufacturer's instructions (Promega, Madison, WI, USA).

### ChIP assay

ChIP was conducted as previously described [52]. MCF-7 cells transfected with the appropriate plasmids were cultured for 24 h. The medium was replaced with phenol red-free medium containing 2% charcoal-stripped fetal bovine serum (Gibico) for 24 h, followed by treatment with or without 10 nM E2 for 16 h. 10% of each chromatin solution was input DNA. Chromatin immunoprecipitation was carried out at 4°C overnight with the appropriate antibodies. The primers used in the ChIP PCR analysis were 5′-GGCCATCTCACTATGAATCACTTCTGC-3′ (forward) and 5′-GGCAGGCTCTGTTTGCTTAAAGAGCG-3′ (reverse) for *pS2* promoter [54].

### Cell growth assays

Cell growth was examined by 3-(4,5-dimethylthiazol-2-yl)-2,5-diphenyltetrazolium bromide (MTT) and crystal violet staining assay. For MTT assay, MCF-7 cells stably transfected with the appropriate plasmids were selected on the basis of resistance to G418 or hygromycin B. The cells were seeded in the appropriate plates and cultured for 24 h. After that, the medium was replaced with phenol red-free medium containing 2% charcoal-stripped fetal bovine serum (Gibco) for 24 h, followed by treatment with or without 10 nM E2 for several days. MTT assay was conducted according to the manufacturer's instructions (Key Gen, Nanjing, China). The absorbance of the samples was determined at 490 nm, and the values were normalized to that of control cells. For crystal violet staining assay, MCF-7 cells stably transfected with the appropriate plasmids were seeded into 35 mm plates. After recognizable clones appeared, the cells were stained with crystal violet for 30 min at room temperature. For quantitative analysis, the stained cells were solubilized in DMSO, and the absorbance was measured at 570 nm [53]. For flow cytometry assay, MCF-7 cells transiently transfected with the appropriate plasmids were cultured in 35-mm plates for 24 h. After that, the medium was replaced with phenol red-free medium containing 2% charcoal-stripped fetal bovine serum (Gibico) for 24 h, followed by treatment with or without 10 nM E2for 16h. The cells were then fixed in anhydrous ethanol at −20°C for overnight. After washing, the cells were stained with propidium iodide (PI) (BD Pharmingen, CA) at 37°C for 30 min. Cell cycle profiles were determined by flow cytometry assay using ModFit LT (BD Biosciences, San Jose, CA, USA). Experimental data were collected by FACSCalibur (BD Biosciences, San Jose, CA, USA).

### RNA extract and RT-PCR

Total RNA was extracted from MCF-7 cells using Takara RNAiso Reagent (Takara, Dalian, China). Total RNA (1 μg) was reverse transcribed using oligo (dT) primer and a Reverse Transcription System (Takara). The single-stranded cDNA was amplified by PCR using specific primers: *Cyclin D1*: 5′-TGGAGGTCTGCGAGGAACAGAA-3′ (forward) and 5′-TGCAGGCGGCTCTTTTTCA-3′ (reverse); *pS2*: 5′-ATGGAGAACAAGGTGATCTG-3′ (forward) and 5′-CCACAATTCTGTCTTTCACG-3′ (reverse); *TCF21*: 5′-AGCTACATCGCCCACTTGAG-3′ (forward) and 5′-CGGTCACCACTTCTTTCAGG-3′ (reverse) and *β-actin*: 5′-TGGAGAAAATCTGGCACCACACC-3′ (forward) and 5′-GATGGGCACAGTGTGGGTGCCC-3′ (reverse). All PCR products were analyzed by 1.5% agarose gel electrophoresis. Each gene was measured in triplicate.

### Statistical analysis

All data analysis was performed with ANOVA, followed by the Bonferroni test for pairwise comparisons [53]. Data were expressed as means ±SDs. Statistical significance was considered at the *P*-value <0.05 or 0.01 level.
